# A Case of Abortion of Triplets

**Published:** 1859-10

**Authors:** N. C. Husted

**Affiliations:** 217 W. 42d Str.


*A case of Abortion of Triplets.*—The previous history of the lady who is the subject of my remarks, may not be uninteresting to the reader:

Mrs. D——, aged 31; born in Ireland, has had six children at the full time, and three miscarriages. The presentations of the six are as follows: 1st, vertex; 2d, vertex; 3d, vertex; 4th, shoulder; 5th, shoulder; 6th, shoulder. The first three children are living; the last three were still-born.

Her first miscarriage was at the second month; the second occurred at or about the third month, and were twins. Her third or last were triplets at the sixth month or thereabouts. The time between the delivery of each fœtus was twenty four hours; the first was born on Sunday evening; the second on Monday evening; the third on Tuesday evening, at about the same time, not varying one hour. The presentations were as follows:—1st, vertex; 2d, breech; 3d, shoulder; two were males and the third was a female. There was no unusual flooding between the deliveries *of each fœtus.*

Her general health had been good up to the time of her miscarriage, except that she had some trouble of mind. The time between the birth of each of these fœtuses is remarkable. It is also remarkable that the two last miscarriages occurred within one year. The woman has recovered her usual strength and vigor, and bids fair to have an increase in her family.

N. C. Husted, M. D.,
217 W. 42d Str.

